# Key Mechanistic Principles and Considerations Concerning RNA Interference

**DOI:** 10.3389/fpls.2020.01237

**Published:** 2020-08-13

**Authors:** Petr Svoboda

**Affiliations:** Institute of Molecular Genetics of the Czech Academy of Sciences, Prague, Czechia

**Keywords:** RNAi, dicer, argonaute, miRNA, dsRNA, off-targeting

## Abstract

Canonical RNAi, one of the so-called RNA-silencing mechanisms, is defined as sequence-specific RNA degradation induced by long double-stranded RNA (dsRNA). RNAi occurs in four basic steps: (i) processing of long dsRNA by RNase III Dicer into small interfering RNA (siRNA) duplexes, (ii) loading of one of the siRNA strands on an Argonaute protein possessing endonucleolytic activity, (iii) target recognition through siRNA basepairing, and (iv) cleavage of the target by the Argonaute’s endonucleolytic activity. This basic pathway diversified and blended with other RNA silencing pathways employing small RNAs. In some organisms, RNAi is extended by an amplification loop employing an RNA-dependent RNA polymerase, which generates secondary siRNAs from targets of primary siRNAs. Given the high specificity of RNAi and its presence in invertebrates, it offers an opportunity for highly selective pest control. The aim of this text is to provide an introductory overview of key mechanistic aspects of RNA interference for understanding its potential and constraints for its use in pest control.

## Introduction

RNA interference (RNAi) is one of the pathways, collectively named RNA silencing pathways, that employ small RNAs as guides for sequence-specific silencing [reviewed in (Ketting, 2011)]. RNAi was discovered in *C. elegans* and defined as sequence-specific mRNA degradation induced by long double-stranded RNA (dsRNA) (Fire et al., 1998). Although some authors use the term RNAi as a synonym for RNA silencing [e.g., (Ketting, 2011)], this review will adhere to the original definition as formulated by Fire et al.

The primary aim of this contribution is to provide an overview of RNA interference mechanism with focus on selected aspects concerning RNAi targeting and off-targeting in animals as these would be most relevant features for discussing the use of RNAi for pest control. Therefore, I will purposefully not go into the details. Interested readers should check out referenced reviews or original articles. For a thorough overview of RNAi, readers are welcome to refer to a comprehensive compilation of information on RNAi and related pathways in different animal taxons and plants, which we assembled with colleagues for the European Food and Safety Authority (Paces et al., 2017).

## Principles of RNA Silencing and Common Denominators

**Figure 1:**
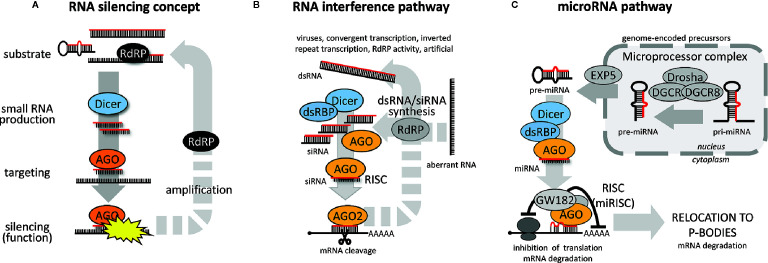
RNA silencing pathways. **(A)** General concept of RNA silencing. **(B)** General RNAi pathway overview, and **(C)** miRNA pathway (animal set up).

Some kind of RNA silencing pathway (**Figure 1A**) exists in almost every eukaryotic organism with some notable exceptions among fungi and protists (Nakayashiki et al., 2006; Matveyev et al., 2017). RNA silencing pathways utilize 20-30 nucleotide long RNAs loaded on Argonaute proteins, which guide sequence-specific repression through basepairing with target RNAs. RNA silencing pathways differ in the origin and biogenesis of small RNAs, mechanisms leading to target repression, and biological roles [reviewed in (Ketting, 2011)].

RNA substrates giving rise to small RNA guides in RNA silencing pathways vary in structure. They include double-stranded RNA (dsRNA) with blunt ends, small and long RNA hairpins with perfect and less-than-perfect complementarity, sense and antisense RNA (basepaired or not), or single-stranded “aberrant” RNA that would be converted to dsRNA by an RNA-dependent RNA polymerases (RdRP) or converted directly to small RNAs. Substrates can be converted to a small RNA either by Dicer, an RNase III cleaving dsRNA and/or canonical microRNA (miRNA) precursors, or by some Dicer-independent mechanism [reviewed in (Kim et al., 2009)].

Target repression can be post-transcriptional or transcriptional. Post-transcriptional RNA silencing could have a form of endonucleolytic cleavage of cognate RNA (traditionally associated with RNAi), or translational repression coupled with mRNA destabilization (historically associated with animal miRNAs). Transcriptional RNA silencing is common in plants but rare among animals [reviewed in (Wassenegger, 2005; Malik and Svoboda, 2012)]. It may involve *de novo* DNA methylation or transcriptionally repressive histone modifications.

Common biological roles of RNA silencing pathways include regulation of endogenous gene expression, antiviral immunity, and genome protection against transposable elements [summarized in (Ketting, 2011)]. During evolution, RNA silencing could evolve into a complex system of interconnected pathways [exemplified by plants, reviewed for example in (Borges and Martienssen, 2015)] or into a relatively simple set up (mammalian soma). The following text will focus on RNAi but includes also the miRNA pathway because of its close mechanistic relationship to RNAi.

## RNAi Pathway

The canonical RNAi pathway (**Figure 1B**) is initiated by cleavage of long dsRNA into small interfering RNAs (siRNAs). One siRNA strand then becomes loaded onto an Argonaute protein possessing endonucleolytic activity (e.g., AGO2 in vertebrates and arthropods). A complementary mRNA is cleaved by the Argonaute in the middle of the siRNA:mRNA duplex. In some taxons (e.g., plants or *C. elegans*), RNAi pathways employ the above-mentioned RdRPs, which can provide an amplification loop synthesizing small RNAs or dsRNA on targeted RNA templates [reviewed in (Maida and Masutomi, 2011)]. *C. elegans* employs so-called “transitive RNAi” where RdRP produces secondary siRNAs extending upstream of the targeted sequence (Sijen et al., 2001). Plants also exhibit transitive silencing (Vaistij et al., 2002); the transitivity may even spread downstream of the targeted sequence (Moissiard et al., 2007).

Canonical RNAi is traditionally viewed as a defense pathway providing antiviral innate immunity in invertebrates and plants against viruses that produce dsRNA (Ding and Voinnet, 2007). However, RNAi could evolve additional roles, such as maintenance of genome integrity through suppression of transposable elements or control of gene expression. In plants, for example, the basic RNAi mechanism has been integrated into a complex pathway system of post-transcriptional and transcriptional silencing, which employs multiple Dicer, Argonaute and RdRP proteins and functions in antiviral defense, protection of genome integrity, and regulation of gene expression [reviewed for example in (Bologna and Voinnet, 2014; Borges and Martienssen, 2015)]. In *C. elegans*. RNAi exists as a complex of antiviral RNAi, endo-RNAi controlling endogenous genes, and exo-RNAi responding to dsRNA in the environment [reviewed in (Billi et al., 2014)]. RNAi is functional in insects (Dowling et al., 2016) and other arthropod subphyla, including *Chelicerata* [ticks and mites (Kurscheid et al., 2009; Schnettler et al., 2014; Hoy et al., 2016)] and *Crustacea* [shrimps (Chen et al., 2011; Huang and Zhang, 2013; Yang et al., 2014)]; genomic data suggest that *Myriapoda* arthropods also have functional RNAi (Palmer and Jiggins, 2015). In vertebrates, the RNAi pathway has become vestigial; protein factors for siRNA biogenesis and target repression serve the miRNA pathway [reviewed in (Svoboda, 2014)]. This is presumably a consequence of the innate immunity system evolving an array of protein sensors detecting pathogen markers such as dsRNA, which trigger the so-called interferon response [reviewed in (Gantier and Williams, 2007)]. An important limiting factor for functional RNAi in somatic mammalian cells seems to be inefficient siRNA production due to the low processivity of mammalian Dicer, which is adapted for non-processive miRNA biogenesis (Demeter et al., 2019).

## miRNA Pathway

While the miRNA pathway (**Figure 1C**) can share some components with the RNAi pathway, it differs in several fundamental aspects. miRNAs are genome-encoded repressors of gene expression with defined sequences (i.e., can be precisely annotated). While RNAi employs a population of siRNAs stochastically generated from dsRNA to destroy a pool of RNAs with the complementary sequence, one specific miRNA sequence can guide repression of many different mRNAs through imperfect miRNA:mRNA basepairing.

Animal miRNA biogenesis [reviewed in (Kim et al., 2009)] starts with a primary miRNA (pri-miRNA), a long Pol II transcript carrying one or more local hairpins, which can be cut out from the pri-miRNA by RNase III activity of the nuclear Microprocessor complex. The resulting miRNA precursor (pre-miRNA) is transported to the cytoplasm, where it is cleaved by Dicer. One strand of the resulting duplex is loaded onto an AGO protein similarly to the RNAi pathway. Vertebrates have usually four AGO paralogs; teleost fish acquired an additional AGO3 paralogue through a fish-specific genome duplication event (Mcfarlane et al., 2011). All four mammalian AGO proteins accommodate miRNAs equally well (Meister et al., 2004), including AGO2, which is the only one with “slicing” endonucleolytic activity. All four mouse AGO proteins seem to be functionally redundant in the miRNA pathway, as shown by rescue experiments in embryonic stem cells lacking all four *Ago* genes (Su et al., 2009).

Typical miRNA:mRNA interaction in animals occurs with partial complementarity (described in detail further below) and results in translational repression, which is associated with substantial mRNA degradation. Plant miRNA biogenesis [reviewed in (Jones-Rhoades et al., 2006)] employs one of the Dicer paralogs (DCL1), which processes both pri-miRNA and pre miRNA. Plant miRNAs often have higher sequence complementarity resulting in RNAi-like cleavage of their targets but also frequently repress translation (Brodersen et al., 2008; Lanet et al., 2009). In animals, miRNAs can also mediate RNAi-like cleavage, as demonstrated by reporters designed to have full complementarity to a specific miRNA (Schmitter et al., 2006), but naturally occurring RNAi-like endonucleolytic cleavage of targets is rare (Yekta et al., 2004). The experimental approach to knocking down gene expression in mammalian cells by delivering a siRNA (either as an *in vitro* synthesized RNA or expressed from a plasmid vector) is commonly called RNAi. Mechanistically, however, the approach hijacks the miRNA pathway and its aforementioned ability to produce RNAi-like cleavage.

## Co-Existence of RNAi and miRNA Pathways

**Figure 2:**
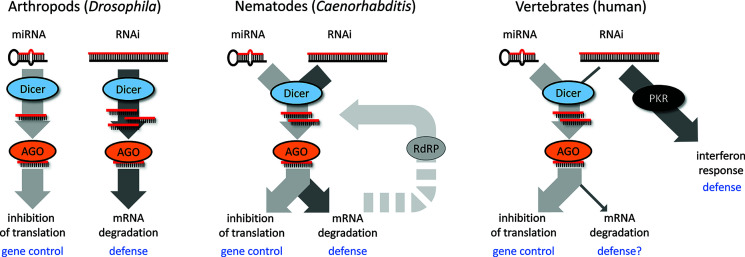
Different scenarios of co-existence of RNAi and microRNA (miRNA) in different species.

While there is an apparent mechanistic overlap, there is functional divergence of RNAi and miRNA pathways, which likely influenced the co-existence of the two pathways in different model systems during evolution (**Figure 2**). One is represented by *Drosophila*, where both pathways genetically diverged such that each pathway has a dedicated Dicer and AGO protein, while the crosstalk between the two pathways is minimal. Dicer in the RNAi pathway is phylogenetically more derived, which would be consistent with its engagement in dsRNA-based antiviral defense and host-pathogen evolutionary arms race (Murphy et al., 2008; Obbard et al., 2009). *C. elegans* employs a single Dicer in production of miRNAs and siRNAs, but has a complex system of Argonaute proteins and RdRP amplification, which contributes to the separation of the pathways. Mammals have a single Dicer mainly serving for miRNA biogenesis; canonical RNAi was functionally replaced by the interferon response, which allows for sensing more structural features of replicating RNA viruses. Functional RNAi in mammalian cells requires high Dicer activity, enough dsRNA substrate, and suppression of the interferon response (Kennedy et al., 2015; Maillard et al., 2016; Kennedy et al., 2017; Van Der Veen et al., 2018; Demeter et al., 2019). However, these three conditions are rarely met—a unique example occurs in the mouse oocyte [reviewed in (Svoboda, 2014)].

Interestingly, in one of the plant RNA silencing mechanisms, RNAi essentially serves as an amplifier of miRNA silencing where miRNA-mediated cleavage of mRNA targets is followed by RdRP-mediated production of long dsRNA, which is processed by Dicer into so-called phased siRNAs (phasiRNA). PhasiRNAs themselves are a complex small RNA category as they can be generated by different Dicers and mediate target cleavage as well as transcriptional silencing. (reviewed in (Komiya, 2017; Deng, 2018).

## Important Mechanistic Details of RNAi

### Substrate Processing by Dicer and Types of Small RNA Populations

**Figure 3:**
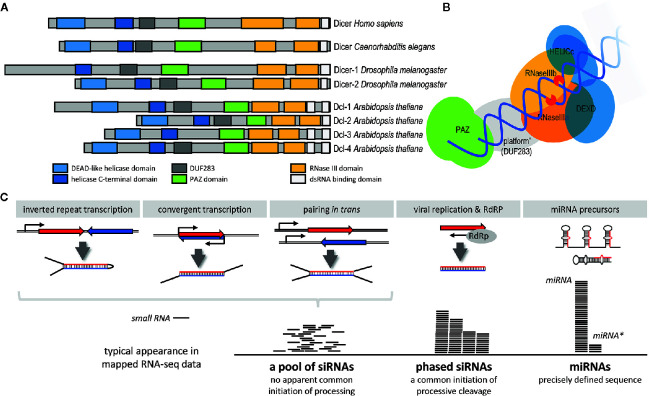
Dicer and production of small RNAs. **(A)** Domain composition of Dicer proteins in a common multicellular model system. **(B)** Schematic organization of Dicer and its interaction with dsRNA [based on the mammalian Dicer structure (Lau et al., 2012)]. **(C)** Model examples of different types of Dicer substrates and products. Production of phased small interfering RNAs (siRNAs) requires a double-stranded (dsRNA) terminus where Dicer will initiate processive cleavage. It could be produced by an RdRP, typically during viral replication. In specific cases, such as plant phased siRNAs (phasiRNA), also by a cellular RdRp [reviewed in (Komiya, 2017)]. However, not all RdRP-produced dsRNAs result in the formation of phased RNAs.

RNase III Dicer (reviewed in detail in [Jaskiewicz and Filipowicz, 2008; Svobodova et al., 2016)] is the enzyme producing small RNAs in canonical RNAi and miRNA pathways. Dicer is a large (~200 kDa) multidomain protein (**Figure 3A**). Structural and biochemical analyses (mainly in mammals but also in the protozoan *Giardia intestinalis*) uncovered how canonical Dicer generates small RNAs of defined length from long dsRNA substrates (Provost et al., 2002; Zhang et al., 2002; Zhang et al., 2004; Macrae et al., 2006; Macrae et al., 2007). Dicer preferentially cleaves dsRNA at the termini (**Figure 3B**). A dsRNA terminus is bound by the PAZ domain, which has high affinity to 3’ protruding overhangs, typical termini of canonical miRNA precursors and of processive cleavage of long dsRNA (Lingel et al., 2003; Song et al., 2003; Yan et al., 2003; Ma et al., 2004). A canonical Dicer functions as a molecular ruler defining the length of a small RNA by the distance between the PAZ domain and RNase III cleavage sites (Macrae et al., 2006). Dicer has two RNase III domains, which form a single processing center containing two catalytic “half sites” (Zhang et al., 2004; Macrae et al., 2006). Each of them cleaves one strand of the dsRNA, producing a small RNA duplex with two nucleotide 3’ overhangs and 5’ monophosphate and 3’ hydroxyl groups at the RNA termini (Zhang et al., 2004). The length of the product depends on the specific Dicer. A typical length of an animal Dicer product is 22 nucleotides although 20-22 nt siRNAs was reported for different insects (Santos et al., 2019). *Giardia* produces 25 nt small RNAs, plants, which utilize several Dicer paralogs (**Figure 3A**), produce shorter (21/22 nt) and longer (24 nt) small RNAs (Jaskiewicz and Filipowicz, 2008).

Dicer can process structurally different dsRNA substrates—e.g., small hairpins of pre-miRNAs, dsRNA with blunt ends, or dsRNA with long single-strand overhangs or loops (**Figure 3C**). As mentioned above, Dicer structure implies that Dicer preferentially cleaves dsRNA at the termini. However, as shown for human Dicer, it can also cleave the dsRNA stem internally, albeit with low efficiency (Provost et al., 2002; Zhang et al., 2002). The type of dsRNA processing determines the composition of a small RNA population produced from each type of the template (**Figure 3C**). miRNAs are precisely defined because precursors have a uniform structure and there is just a single Dicer cleavage event. Long blunt-end dsRNA, which is cleaved processively from its ends, generates phased siRNAs produced by consecutive cleavage. In this case, there may be some variability/shifts as the termini are not as precisely defined as 2nt overhangs of miRNA precursors. Dicers with low processivity, exemplified by mammalian Dicers, generate siRNAs mainly from dsRNA termini—RNAi efficiency in this case thus depends on the efficiency of the first siRNAs at the termini (Demeter et al., 2019). When Dicer cannot initiate cleavage from a terminus because it is, for example obstructed by longer overhangs, dsRNA processing is initiated by an internal cleavage; the resulting siRNA population appear random and there would be no evidence of phasing [e.g., (Tam et al., 2008; Watanabe et al., 2008)].

### Loading—Small RNA Sorting Onto Argonaute Proteins

Loading of a small RNA onto an Argonaute protein is the key step in formation of the RNAi effector complex also known as RNA-induced silencing complex (RISC). While Argonaute proteins interact with many other proteins [reviewed in (Meister, 2013)], the minimal RNAi effector complex, the holo-RISC, is a specific Argonaute loaded with a siRNA. Loading is an important step for selecting the targeting strand and sorting small RNAs into distinct RNA silencing pathways. As shown for animal Argonautes, loading a specific strand of the small RNA duplex produced by Dicer, exhibits a thermodynamic bias where the strand whose 5′-end is less thermodynamically stable is preferentially loaded onto AGO as the guide strand (Khvorova et al., 2003; Schwarz et al., 2003). This feature is important for designing effective siRNAs for experimental repression.

Loading is assisted by a family of proteins with tandemly organized dsRNA binding domains (dsRBDs), which interact with Dicer and AGO proteins to form the RISC loading complex (RLC). Sorting through RLC varies among animal taxons. For example, *C. elegans* employs a single Dicer protein, but evolved an extreme diversity of Argonaute proteins among common model systems [25-27 Argonaute family members (Buck and Blaxter, 2013)]. Together with RdRPs, RNA silencing in *C. elegans* is a complex system of biogenesis and sorting of primary and secondary cytoplasmic and nuclear small RNAs in soma and germline (Yigit et al., 2006; Buck and Blaxter, 2013). The exo-RNAi pathway in *C. elegans* involves loading of AGO protein RDE-1 with primary siRNAs with the assistance of dsRBP RDE-4 (Tabara et al., 1999; Parrish and Fire, 2001; Tabara et al., 2002; Lu et al., 2005; Wilkins et al., 2005). This is followed by biogenesis of secondary siRNAs (22G RNAs) loaded on AGO protein CSR-1 (Aoki et al., 2007). *C. elegans* miRNAs are exclusively loaded on ALG-1/2 AGO proteins (Correa et al., 2010). *Drosophila* employs dedicated Dicer and Argonaute proteins for RNAi (DCR-2 and AGO2) and miRNA pathways (DCR-1 and AGO1). Loading of each AGO is assisted by two dsRBPs: R2D2 [its orthologs exist in winged insects (Dowling et al., 2016)] is coupled with the RNAi pathways and Loquacious (LOQS) primarily with the miRNA pathway; these two dsRBPs thus bridge processing of specific substrates by both Dicers and their loading onto specific AGO proteins, although the separation is not complete (Forstemann et al., 2007; Tomari et al., 2007; Okamura et al., 2008; Czech et al., 2009; Okamura et al., 2009; Ghildiyal et al., 2010). Mammals, in contrast, have minimal if any sorting of small RNAs and load them onto all four AGO proteins equally well (Meister et al., 2004; Burroughs et al., 2011; Dueck et al., 2012). This is presumably because the mammalian RNAi pathway is vestigial and the silencing machinery primarily serves the miRNA pathway.

### Targeting—The Seed Sequence

**Figure 4:**
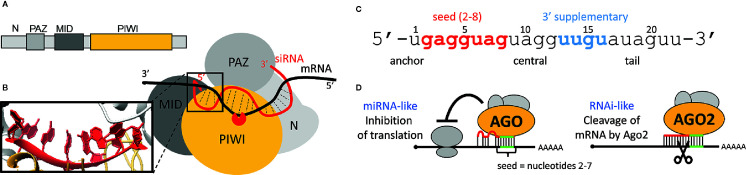
Argonaute protein and target repression. **(A)** Domain composition of human AGO2. **(B)** Schematic organization of domains in AGO2. Magnified is the 5’ end of a small RNA and its A-like form. **(C)** Division of a small RNA into five different modules as described by Wee at al. (Wee et al., 2012). **(D)** Schematic depiction of miRNA-like and RNAi-like silencing effects. An RNAi-like effect requires extensive sequence complementarity and AGO2.

Recognition of targets is coupled with the loaded Argonaute structure (**Figures 4A, B**). The human AGO2 has a bilobed composition with a central cleft for binding guide and target RNAs (Elkayam et al., 2012; Schirle and Macrae, 2012; Schirle et al., 2014; Schirle et al., 2015). AGO2 binds both ends of a siRNA. The 5’ end is buried in a pocket between MID and PIWI domains, while the 3’ end is anchored in the PAZ domain (Ma et al., 2004). The PIWI domain has an RNase H-like fold and provides the endonucleolytic “slicer” activity (Song et al., 2004; Yuan et al., 2005).

A small RNA loaded onto an animal AGO protein has five distinct sequence modules: the anchor, seed, central, 3’ supplementary, and tail (**Figure 4C**) (Wee et al., 2012). The 5’ end nucleotides 2 to 6 are positioned in an A-form (**Figure 4B** - inset) conformation facilitating basepairing with the target (Schirle and Macrae, 2012). Structural analysis of the human AGO2 suggested a stepwise mechanism for interaction with cognate RNAs, where AGO2 exposes nucleotides 2 to 5 for initial target pairing, which then promotes conformational changes that expose nucleotides 2 to 8 and 13 to 16 for further target recognition (Schirle et al., 2014). Structural data were corroborated by kinetic data and single molecule analyses, which support the idea that different regions of the siRNA play distinct roles in the cycle of target recognition, cleavage, and product release (Haley and Zamore, 2004; Li et al., 2012; Wee et al., 2012; Zander et al., 2014; Salomon et al., 2015). The seed sequence disproportionately contributes to target RNA-binding energy, whereas base pairs formed by the central and 3’ regions of the siRNA provide a helical geometry required for catalysis (Haley and Zamore, 2004). Because of the A conformation of the seed, a loaded AGO2 exhibits kinetic properties more typical of RNA-binding proteins and does not follow the rules by which sole oligonucleotides find, bind, and dissociate from complementary nucleic acid sequences (Salomon et al., 2015). Importantly, the concept of the seed sequence is fundamental for understanding one of the main causes of off-targeting.

### Targeting—Complementarity and Cleavage

For the Argonaute function in RNAi, a “two-state” model was proposed (Tomari and Zamore, 2005), where the seed guides binding to the target, while pairing of the 3’ end requires dislodging of the 3’ end from the PAZ domain in order to cleave the cognate RNA. Efficient cleavage requires full complementarity in the middle of the basepaired sequence, in order to be cleaved by the PIWI domain (**Figure 4B**). Mismatches in the central part of the small RNA interfere with the cleavage and explain the high specificity of RNAi (i.e., endonucleolytic cleavage by the AGO2 slicer activity) (**Figure 4D**).

Single-molecule experiments with the loaded AGO2 showed that target binding starts at the seed region of the small RNA (Chandradoss et al., 2015; Jo et al., 2015a; Jo et al., 2015b). AGO2 initially scans for target sites with complementarity to nucleotides 2–4 of the miRNA. This initial interaction propagates into stable association when the target complementarity extends to nucleotides 2–8. The recognition process is coupled to lateral diffusion of AGO2 along the target RNA, which promotes the target search by enhancing the retention of AGO2 on the RNA (Chandradoss et al., 2015). RISC binding with the seed match can thus be established, which is consistent with the seed-match rule of miRNA target selection (Chandradoss et al., 2015; Jo et al., 2015a; Jo et al., 2015b). An important conclusion from the kinetic analysis by Wee et al. is that low-abundant miRNAs are unlikely to contribute biologically meaningful regulations, because they are present at concentrations below their K_D_ for seed-matching targets, which are in a picomolar range (3.7 pM for mouse AGO2 and 20 pM for *Drosophila* AGO2) (Wee et al., 2012). Importantly, accessibility of the target for seed sequence binding is another important factor for efficient targeting. It was shown that the accessibility of the target site correlates directly with the efficiency of cleavage, recognition of inaccessible sequences is impaired because RISC does not unfold structured RNA (Ameres et al., 2007).

siRNA-mediated target recognition is highly specific. However, discrimination of RNAi between two sequences differing by a single nucleotide depends on the position and type of the mismatch (Du et al., 2005; Holen et al., 2005; Haley et al., 2010). Analysis of minimal siRNA complementarity in *Drosophila* showed that perfect complementarity at positions 2–17 is sufficient for RNAi (Haley et al., 2010). G:U wobble basepairs are surprisingly well tolerated; target sites containing such mismatches were silenced almost as efficiently as with full complementarity (Du et al., 2005). Tolerated can also be A:C mismatches (Du et al., 2005).

Of note is that consensus basepairing rules for functional plant miRNA-target interactions differ from those for animals: there is little tolerance of mismatches at nucleotides 2–13, with especially little tolerance of mismatches at nucleotides 9–11, and more tolerance of mismatches at nucleotides 1 and 14–21 (Wang et al., 2015). Furthermore, the perfect complementarity is not as prevalent as usually thought among plant miRNAs, as most of the identified miRNA targets in plant cells have some imperfect basepairing [summarized in (Jones-Rhoades et al., 2006)].

### RdRP Enhancer of RNAi—Transitive RNAi

RdRPs can contribute to RNAi by converting single-stranded RNA to dsRNA or by synthesizing short RNAs that could be loaded onto AGO proteins. Importantly, all RdRPs identified so far seem to come from one ancestral RdRP, whose orthologs were found in plants, fungi and some animals (Cerutti and Casas-Mollano, 2006; Murphy et al., 2008). Homologs of RdRPs exist in numerous metazoan taxons, including *Nematoda* (e.g., *Caenorhabditis elegans*), *Cnidaria* (hydra), *Chelicerata* (tick), *Hemichordata* (acorn worm), *Urochordata* (sea squirt), but appear absent in the genomes of others, including *Platyhelminthes* (planaria), *Hexapoda* (*Drosophila*), or *Craniata* (vertebrates). Consequently, transitive RNAi generating secondary sequences upstream of the region targeted by siRNAs was not observed in *Drosophila* or mouse (Schwarz et al., 2002; Roignant et al., 2003; Stein et al., 2003). Therefore, the absence of an RdRP gene in the genome can help as an indicator of the absence of the amplification loop.

### Environmental and Systemic RNAi

It was shown in pioneering experiments in *C. elegans* that RNAi can be induced by simply soaking the worm into dsRNA solution (Tabara et al., 1998) or feed it bacteria expressing dsRNA (Timmons and Fire, 1998). These spectacular effects combined two distinct phenomena: (i) environmental RNAi where cells can uptake long dsRNA or small RNAs from the environment, and (ii) systemic RNAi where silencing can spread across cellular boundaries. While both phenomena can co-exist in one species, they might be distinct because the RNAi mediator spreading across cellular boundaries can be a different RNA molecule that the original inducing RNA molecule taken up from the environment. As the biology of systemic and environmental RNAi is complex and beyond the scope of this contribution, readers can look for more details into reviews on this topic, such as (Whangbo and Hunter, 2008; Huvenne and Smagghe, 2010; Ivashuta et al., 2015).

dsRNA can be taken up *via* specific transmembrane channel mediated uptake (e.g., *C. elegans* or flower beetle) or through alternative endocytosis [e.g., in *Drosophila*, reviewed in more detail in (Whangbo and Hunter, 2008)]. Non-cell autonomous RNAi has been reported from parasitic nematodes (Geldhof et al., 2007), hydra (Chera et al., 2006), planaria (Newmark et al., 2003; Orii et al., 2003), some insects (Tomoyasu et al., 2008; Xu and Han, 2008), and plants (Himber et al., 2003). Some of the molecular mechanisms underlying systemic and environmental RNAi have been identified, such as dsRNA-transporting channels encoded by *sid-1* and *sid-2* genes (systemic RNAi-deficient), which function in systemic and environmental RNAi in *C. elegans* (Winston et al., 2002). *Sid-1* encodes a conserved transmembrane protein that forms a dsRNA channel and has homologs (but not necessarily orthologs) in a wide range of animals, including mammals (Feinberg and Hunter, 2003; Tomoyasu et al., 2008; Shih et al., 2009; Shih and Hunter, 2011; Cappelle et al., 2016). In contrast, *Sid-2*, which encodes a transmembrane protein, has only been found in several *Caenorhabditis* species (Winston et al., 2007; Dalzell et al., 2011).

In organisms displaying environmental and systemic RNAi, delivery of dsRNA could be used to intervene or harm. This phenomenon underlies strategies for crop protection [reviewed in more detail, for example, in (Cai et al., 2018)] and further discussed in the section *Horizontal Transfer of Small RNAs and RNAi Across Kingdoms*.

Notably, dsRNA itself has a potential to be used directly without producing a transgenic plant – as shown, for example, by topical application of dsRNA, which protected *Nicotiana benthamiana* and cowpea against infection with the potyvirus bean common mosaic virus (Worrall et al., 2019) and other cases [e.g., (Konakalla et al., 2019; Namgial et al., 2019)]. On a large scale, dsRNA feeding was used, for example, to protect bees against acute paralysis virus (Hunter et al., 2010), and spraying dsRNA solution was used to protect plants against fungus *Fusarium graminearum* (Koch et al., 2016).

## Off-Targeting Considerations

One of the frequently raised questions is how specific and selective gene targeting by RNAi is. There is not a simple answer to that question, because there are several different strategies to induce RNAi and each of them has different potential for inducing off-targeting, i.e., downregulating an unintended target. Off-targeting was typically discussed as non-specific effects within one experimental model system [e.g., (Echeverri et al., 2006; Svoboda, 2007)]. In case of RNAi-mediated pest control, off-targeting would mainly consider effects on gene expression in other species than the targeted pest. There are two possible general effects on non-targeted species: (i) RNAi (typically siRNA-based) would induce miRNA-like repression of genes whose transcripts have complementarity to the seed sequence (wrong genes silenced in wrong species), (ii) RNAi would target gene(s) with high sequence similarity to dsRNA/siRNA (right gene (or its homologs) silenced in wrong species).

In addition, off-targeting in mammalian cells was also linked with a sequence-independent interferon response induced by long dsRNA. Although it is not clear whether environmental exposure to doses of dsRNA used for pest control would induce the interferon response in humans or other mammals, it is a testable and resolvable issue.

### miRNA-Like Off-Targeting Effects in Other Species

miRNA-like off-target repression is a common off-targeting issue, particularly troubling RNAi experiments in mammalian cells, where it was shown that the off-target gene repression depends on the siRNA concentration and seed sequence (Jackson et al., 2003; Jackson et al., 2006). Several strategies have been proposed for achieving more selective RNAi in mammalian cells, including good experimental design (e.g., using the lowest effective siRNA concentration and employing specificity controls) or using RNAi-inducing agents with increased specificity—these include (i) chemical modifications eliminating activity of the “passenger”(non-targeting) siRNA strand or affecting seed pairing (Jackson et al., 2006; Chen et al., 2008; Fluiter et al., 2009; Snead et al., 2013; Seok et al., 2016), and (ii) pools of more different siRNAs with the molarity of each seed sequence proportionally diluted. Accordingly, when considering this “miRNA-like” type of off-targeting, the two key factors are the mechanism of RNAi induction and the concentration of the RNAi-inducing molecule (leaving aside additional issues like small RNA sorting into different RNA silencing pathways and a varying crosstalk between miRNA and RNAi in different organisms). In general, a long dsRNA, which is converted into a siRNA pool or a pool of chosen siRNAs, principally represents a low if any risk of miRNA-like off-targeting in contrast to a single targeting siRNA (Stein et al., 2005; Hannus et al., 2014). However, exceptions may emerge: an RNAi screen with long dsRNA in *Drosophila* showed that some long dsRNA sequences yielded off-targeting, which stemmed from short tandem repeat sequences in the dsRNA (Ma et al., 2006).

### Undesirable RNAi Effects in Non-Target Species

This issue is represented by targeting a homologous gene because of existing sequence similarity. This off-target effect is most likely to appear in closely related species in the environment treated with RNAi-based pest control. However, it is difficult to predict at which point the sequence divergence will render RNAi non-effective. As discussed above, a single nucleotide mismatch may be sufficient to prevent targeting, but this depends on the position and type of the mismatch (Du et al., 2005). Given the inhibitory effects of mismatches in the seed sequence and in the central part, 90% sequence identity with evenly distributed mismatches of an off-target homologous gene could be sufficiently diverged to lack perfect complementarity regions >17 nt. The effects on the off-target homolog would also depend on the concentration of RNAi-inducing agent; in one case in *C. elegans*, an 80% sequence identity of two genes yielded cross-interference, which was remedied by reducing concentration of microinjected dsRNA from 1 mg/ml to 100 μg/ml (Tabara et al., 1998). In a study of targeting a V-ATPase gene in the western corn rootworm gene with dsRNA, a silencing of its ortholog in the Colorado potato beetle (80% sequence identity) was observed but LC50 values showed a ten-fold difference in activity (Baum et al., 2007). Analysis of ten insect families in four different orders showed that the dsRNA targeting the *Snf7* gene in western corn rootworm was only active in a subset of species in the *Chrysomelidae* family (leaf beetles) whose *Snf7* genes had >90% identity with the dsRNA sequence (Bachman et al., 2013). While percentage of the sequence identity may be an arbitrary factor as the distribution of mismatches in the sequence is also important, these numbers imply that 80%–90% sequence identity is around threshold for functional RNAi.

An additional important factor is how large the off-target gene downregulation will manifest as a biologically relevant off-target phenotype. RNAi-mediated silencing of gene homologs in other species will likely be less efficient than downregulation of the desired target, because the same amount of dsRNA will produce less functional siRNAs in non-targeted species. Therefore, while off-targeting may be detectable by qPCR, it could be tolerated without adverse effects.

## Horizontal Transfer of Small RNAs and RNAi Across Kingdoms

In 2012, a study suggested that miRNAs from ingested plants could traverse into the mammalian bloodstream and suppress genes in the liver (Zhang et al., 2012). The report received a lot of attention and spurred a major debate because of implications these data could have. We reviewed this issue in detail in the aforementioned report (Paces et al., 2017), including three problematic areas that lacked strong experimental support: (i) the mechanism of transport from the digestive system through the bloodstream to the cells, (ii) the effector complex structure, particularly its loading with single-stranded methylated plant miRNA, (iii) the targeting stoichiometry consistent with the above-mentioned picomolar range of miRNA K_D_. Some of the follow-up studies supported the existence of functionally relevant “xenomiRs” in humans and other mammals, while other studies questioned or rejected the idea (Paces et al., 2017). A recent survey of 824 datasets from human tissue and body fluids argues that human xenomiRs are likely artifacts (Kang et al., 2017). Among the strong arguments against biologically relevant dietary xenomiRs in humans were: the minimal fraction of xenomiRs (0.001% of host human miRNA counts), apparent batch effects of xenomiRs, no significant enrichment in sequencing data from tissues and body fluids exposed to dietary intake (e.g., liver), no significant depletion in tissues and body fluids that are relatively separated from the main bloodstream (e.g., brain and cerebro-spinal fluid), and, remarkably, the observation that the majority (81%) of body fluid xenomiRs would stem from rodents, an unlikely dietary source but common experimental material. These data argue that miRNAs from the diet are not uptaken by mammals and integrated into their miRNA pathways. At the same time, organisms with environmental and systemic RNAi can be susceptible to dietary uptake of dsRNA or small RNA. This was already shown in the pioneering RNAi experiments mentioned above – soaking in dsRNA or feeding dsRNA-expressing bacteria could suppress gene expression in *C. elegans* (Tabara et al., 1998; Timmons and Fire, 1998).

Consequently, trans-kingdom RNAi potential could be exploited in plants expressing dsRNA and selectively targeting RNAi-sensitive pests with an outcome of choice, e.g., repelling the pest, immobilizing it, sterilizing it (Bhatia et al., 2012), or killing it (Baum et al., 2007; Mao et al., 2007; Bhatia et al., 2012; Zhang et al., 2015; Kola et al., 2016). Processing of expressed dsRNA by plant’s RNA silencing machinery, which could reduce amount of dsRNA ingested by a pest or cause off-targeting of plant genes, can be prevented by localizing dsRNA expression into chloroplasts (Zhang et al., 2015). Given the genome sequence diversity and relatively high sequence specificity of RNAi, an RNAi-based pesticide could represent a biodegradable, highly selective pesticide with an adjustable selectivity for the pest control [reviewed, for example, in (Kunte et al., 2020)].

Every new technology brings safety concerns. If the small RNAs can spread, could an RNAi-inducing transgene in a plant or topical application of dRNA/siRNA also affect a non-targeted organisms? What could be the consequences? In principle, the off-targeting risk is inherent to the RNAi approach, but it can be monitored and significantly reduced by a proper experimental design. Furthermore, if RNAi were induced transiently (i.e., through dsRNA or siRNA), the transient nature of RNAi would allow recovery from the off-targeting within days in the species lacking an RdRP amplification loop producing secondary siRNAs. It could take longer if the off-targeting triggered transitive RNAi in the species with an RdRP and/or could induce transcriptional silencing. Transgenerational silencing [reviewed in (Rechavi and Lev, 2017)] has variable duration. In *C. elegans*, RNAi targeting genes expressed in the soma typically affects only the F1 progeny, although exceptional transgenerational silencing for up to 13 generations was also reported (Minkina and Hunter, 2017). Importantly, the probability of inducing a long transgenerational off-target effect in an organism other than the targeted one is negligible for dsRNA sequences with good sequence divergence from closely related species.

## Resistance to RNAi

There is always a risk of resistance to RNAi. In the case of an RNAi-based pesticide, one could expect selection for mutations affecting RNAi efficiency rendering the RNAi-based pesticide ineffective. This could either involve accumulation of mutations within the sequence of the pest target gene (rather unlikely for long dsRNA), mutations within RNAi pathway factors of the pest (including uptake mechanisms), or evolution of *bona fide* RNAi suppressor proteins, which are known defense strategy against RNAi used by viruses (Roth et al., 2004; Haasnoot et al., 2007; Nayak et al., 2010).

Animals lacking RNAi may be viable and fertile, as shown in an *rde-1* mutant in *C. elegans* (Tabara et al., 1999). In fact, wild type isolates of *C. elegans* vary in the RNAi response and may exhibit different degrees of resistance to RNAi (Tijsterman et al., 2002; Elvin et al., 2011; Felix et al., 2011) despite the fact that some of the mutations could make them more susceptible to infection (Felix et al., 2011). A similar scenario could be expected for pests targeted by RNAi that would acquire some mutations in the RNAi pathway. Since most mutations would be recessive, the manifestation of resistance (and strong positive selection) would require homozygosity. Evolved resistance against dsRNA was reported in western corn rootworm (Khajuria et al., 2018). It was a single locus recessive mutation resulting in impaired luminal uptake of dsRNA (Khajuria et al., 2018). Therefore, one should consider the reproduction and life cycle of the targeted pest to develop an optimal treatment regimen to reduce (or not facilitate) the probability of occurrence of homozygous RNAi pathway mutants.

## Summary

RNAi offers selective gene targeting in a species-specific manner. RNAi induced by long dsRNA or unmodified siRNA offers a species-specific biodegradable pesticide. RNAi can be a particularly potent tool against pests that display environmental and systemic RNAi. The risk of potential off-targeting effects can be minimized when selecting the target and its sequence. Off-targeting effects can be monitored in closely related species and targets and, if identified, they would disappear after termination of the RNAi treatment.

## Author Contributions

The author confirms being the sole contributor of this work and has approved it for publication.

## Funding

This work was supported by the Ministry of Education, Youth, and Sports project NPU1 LO1419. The content is based on a presentation given at the OECD Conference on RNAi-based Pesticides, which was sponsored by the OECD Co-operative Research Programme: Biological Resource Management for Sustainable Agricultural Systems whose financial support made it possible for the author to participate in the workshop.

## Conflict of Interest

The author declares that the research was conducted in the absence of any commercial or financial relationships that could be construed as a potential conflict of interest.
